# Impact of the lateral skeletal stability following bilateral sagittal split ramus osteotomy for mandibular asymmetry

**DOI:** 10.1016/j.jpra.2023.08.006

**Published:** 2023-08-17

**Authors:** S. Hasegawa, J. Sasaki, H. Nakao, M. Tomimatsu, S. Yamamoto, S. Watanabe, S. Miyabe, H. Miyachi, M. Goto

**Affiliations:** Department of Maxillofacial Surgery, School of Dentistry, Aichi-Gakuin University, Aichi, Japan

**Keywords:** Positional plagiocephaly, Mandibular asymmetry, Stability, Sagittal split ramus osteotomy, Cranial vault asymmetry index

## Abstract

This study evaluated the stability of bilateral sagittal split ramus osteotomy (BSSRO) associated with positional plagiocephaly and temporal and masseter muscles using posteroanterior cephalogram analysis and three-dimensional computed tomography (3D-CT). This retrospective cohort study included 31 patients who underwent BSSRO for mandibular asymmetry. The cranial vault asymmetry index (CVAI) and the cephalic index were used as indicators of positional plagiocephaly. The distance from the vertical reference line to the menton (Me) was measured on posteroanterior cephalograms immediately and 1 year after surgery, and postoperative stability was assessed. Temporal and masseter muscles were constructed from 3D-CT data and their volumes were measured. Simple regression analysis showed a significant correlation between postoperative changes in the vertical reference line to the Me and the CVAI (*R* = 0.56, *p* = 0.001), the amount of surgical movement in the vertical reference line to the Me (*R* = 0.41, *p* = 0.023), and the variable temporal muscle volume (*R* = 0.27, *p* = 0.028). There was no significant correlation between postoperative changes in the vertical reference line to the Me and the cephalic index (*R* = 0.093, *p* = 0.62) and variable masseter muscle volume (*R* = 0.16, *p* = 0.38). According to multivariate analysis, CVAI (*p* = 0.003) and amount of surgical movement in the vertical reference line to the Me (*p* = 0.014) were significant predictors of postoperative change in the vertical reference line to the Me. Positional plagiocephaly and amount of surgical movement influence lateral skeletal stability following BSSRO for mandibular asymmetry.

## Introduction

When performing bilateral sagittal split ramus osteotomy (BSSRO) for facial asymmetry, what indicators do you use to estimate relapse? Is it distance of surgical movement, temporal muscle volume, masseter muscle volume, or cranial vault asymmetry index (CVAI)?

When facial asymmetry is evident, the psychosocial impact on the individual can be profound.^1^ Patients with significant facial asymmetry are generally interested in restoring functional occlusion and improving aesthetics and appearance.^2^ Therefore, patients may find it unacceptable to revert from the symmetric facial appearance achieved after extensive surgery to the asymmetric facial appearance and wish they had never undergone surgery.

Although BSSRO has become increasingly common in recent decades, there is still controversy regarding skeletal lateral stability after orthognathic surgery. Although the amount of mandibular movement, deep bite, facial skeletal type, fixation materials, and surgical method have been reported as potential risk factors for stability after orthognathic surgery, they remain controversial.^3^^,^^4^ Choi et al.^5^ reported that the amount of horizontal and vertical mandibular relapse significantly increased with the amount of mandibular setback.

Previously, we investigated the relationship between positional plagiocephaly and facial asymmetry and found a significant difference in temporal muscle volume based on positional plagiocephaly and facial asymmetry.^6^ These findings suggest that facial asymmetry due to positional plagiocephaly may be related to changes in temporal muscle development. Because orthognathic surgery is used for the treatment of facial asymmetry and positional plagiocephaly is a factor in causing facial asymmetry, we speculated that untreated positional plagiocephaly might affect stability after orthognathic surgery for facial asymmetry. However, no reports have evaluated the relationship between positional plagiocephaly and postoperative stability after orthognathic surgery.

The purpose of this study was to determine whether the stability of orthognathic surgery for mandibular asymmetry is related to positional plagiocephaly, masticatory muscles, and distance of surgical movement using posteroanterior (PA) cephalogram and three-dimensional computed tomography (3D-CT) analyses through a morphologic and anatomic study.

## Materials and methods

### Patients

This study was conducted on 31 patients who underwent BSSRO for mandibular asymmetry at the Department of Maxillofacial Surgery, Aichi-Gakuin University School of Dentistry, between 2012 and 2019. Patients over 18 years of age with lower facial asymmetry and completed mandibular growth were included. Exclusion criteria were as follows: inability to obtain PA cephalogram and 3D-CT data at 1 year after surgery, a history of genioplasty, a deformed mandible on the same side as the oblique head, and hereditary or congenital diseases. Clinical evaluation, PA cephalogram and 3D-CT examination were performed. All patients received preoperative orthodontic treatment, underwent BSSRO, which includes mandibular setback and rotation procedures, and had a 4-hole locking titanium mini-plate fixed bilaterally on the buccal side with four locking screws. In BSSRO, the temporalis muscle is not separated from the muscle process.

The ethics committee of Aichi-Gakuin University approved this retrospective study (approval number 556), and all patients provided written informed consent before participating in the study. All procedures were performed in accordance with the tenets of the Declaration of Helsinki, “Ethical Principles for Medical Research Involving Human Subjects”.

### Posteroanterior cephalogram analysis

Preoperative deviation, postoperative change, and amount of surgical movement of the menton (Me) were analysed using preoperative, postoperative day 1, and postoperative year 1 PA cephalograms. Photographs were taken with bilateral ear-rods, and the film cassette was lightly touched to the nose. The head was positioned so that the plane passing through the inferior margin of the left orbit and the superior margin of both external auditory canals (Frankfurt plane: FH plane) was parallel to the floor. The mandible was positioned in the maximum intercuspal position.

**Figure 1:**
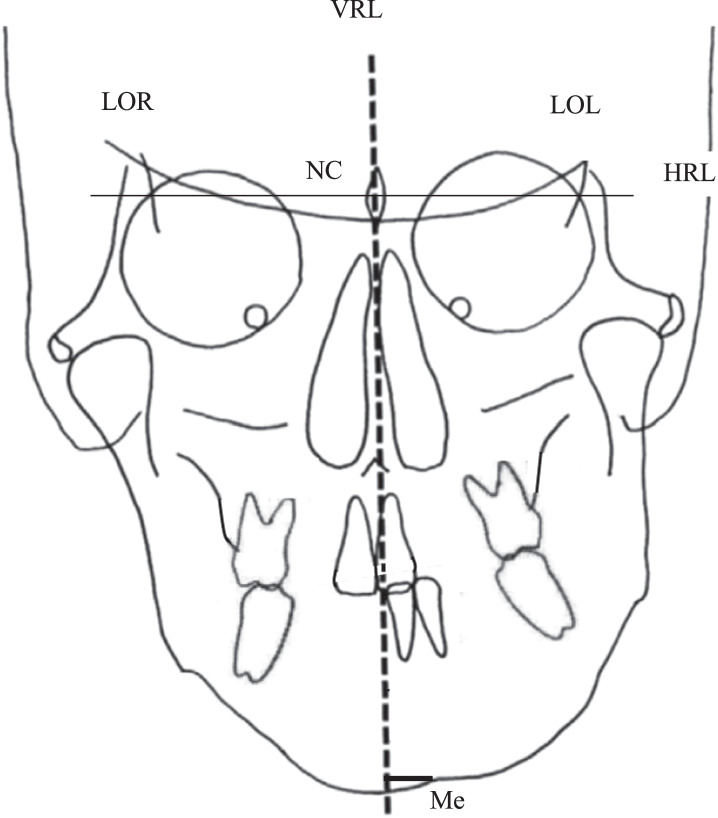
Analysis of symmetry, based on cephalogram data. VRL-Me: distance of the vertical reference line (VRL)-menton (Me). VRL, vertical reference line; Me, menton; LOR, latero-orbitale right; NC, neck of the crista galli; LOL, latero-orbitale left; HRL, horizontal reference line.

PA cephalometric data were stored in Digital Imaging and Communication in Medicine (DICOM) format, transferred to a computer, and processed using A to Z graphic processing software (Yasunaga Computer Systems, Fukui, Japan) to measure each of the reference points shown below. Using the method described by Ricketts,^7^^,^^8^ we analysed the measurement points and frontal cephalometric parameters as shown in Figure 1. The horizontal reference line (HRL) was a straight line connecting the left and right lateral orbits. The vertical reference line (VRL), as the facial midline, passed through the restenosis at the neck of the crista galli (NC), and the line was set orthogonal to the HRL. The line orthogonal to Me was established from the VRL, and the distance from the VRL to Me was measured and defined as the deviation of the menton (VRL-Me).

The amount of change due to surgery was measured as VRL-Me in the pre- and postoperative cephalograms on postoperative day 1 and postoperative year 1; the postoperative change in VRL-Me was assessed by subtracting VRL-Me at 1 year from VRL-Me at 1 day after surgery (Table 1). To clarify the measurements on the left and right sides, negative values were assigned to the right side and positive values to the left side.

**Table 1:** Definition of posteroanterior cephalogram parameters and 3D construction image measurements.

Preoperative deviation of VRL-Me (mm, right side: −, left side: +)	Distance of VRL-Me before surgery
Postoperative change in VRL-Me (mm, absolute value)	Distance of VRL-Me at 1 day after SSRO –-distance of VRL-Me at 1 year after SSRO
Variable temporal muscle volume (mm^3^, right>left: +, right<left: -)	Right temporal muscle volume - left temporal muscle volume
Variable masseter muscle volume (mm^3^, right>left: +, right<left: -)	Right masseter muscle volume - left masseter muscle volume
CI (%)	Cranial index
CVAI (%, prominent right frontal forehead: +, prominent left frontal forehead: -)	Cranial vault asymmetry index

SSRO, sagittal split ramus osteotomy; CVAI, cranial vault asymmetry index; CI, cephalic index; VRL-Me, distance of vertical reference line to the menton.

### Three-dimensional computed tomography analysis to calculate cephalic index, cranial vault asymmetry index, temporal muscle volume, and masseter muscle volume

A three dimentional image of the bone and muscle was reconstructed from helical CT (Asterion Super 4; Toshiba Medical Systems, Tochigi, Japan) data obtained from patients in the supine position. CT images were obtained at the patients' first visit to facilitate surgical treatment planning, and it was assumed that orthodontic treatment and orthognathic surgery had not been performed. CT scans were performed with sequences acquired 0.5 mm apart for 3D reconstructions (120 kV; mean tube current, 150 mA; helical pitch and 3.5). The slice thickness of the reconstructed image was 1.0 mm. CT data were transferred in DICOM format to a DICOM workstation and reconstructed as 3D images using Mimics software (version 19.0, materialise, Leuven, Belgium). The 3D reference planes were set according to the methods described by Katsumata et al.^9^ A single experienced examiner evaluated all 3D datasets.

**Figure 2:**
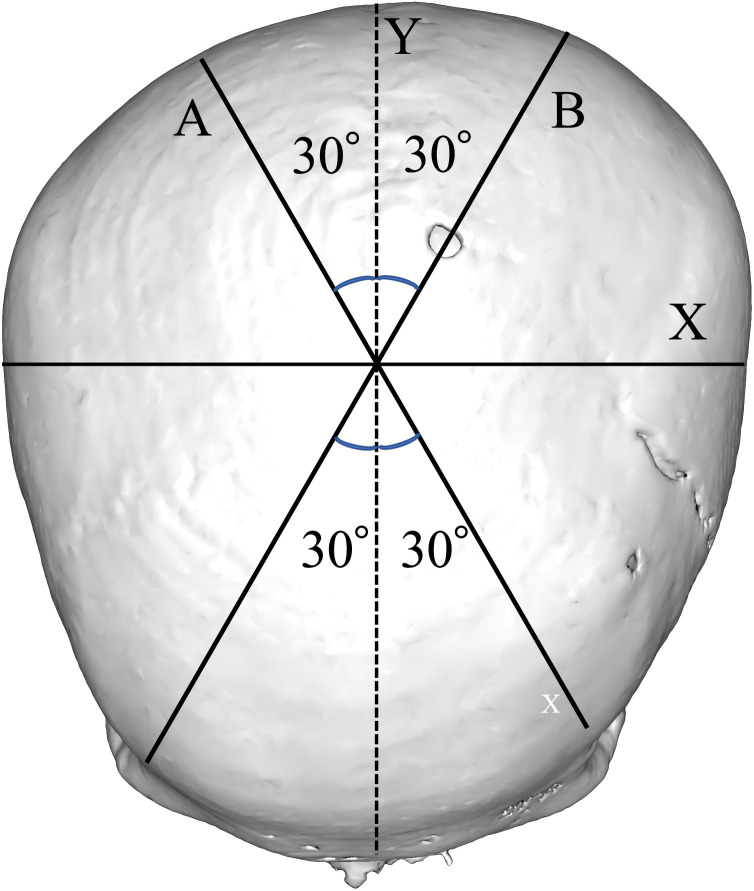
Analysis of positional plagiocephaly, based on 3D-construction image. CI (cephalic index) (%) = X*/Y* × 100, CVAI (cranial vault asymmetry index) (%) = (*A* − *B*) × 100/A or B (whichever is greater). CVAI, cranial vault asymmetry index; CI, cephalic index.

The following symmetry-related variables were used to analyse head shape: cephalic index (CI) and CVAI, both calculated according to the methods described by Loveday and de Chalain.^10^ CI was calculated from head width and length measurements according to the equation shown in Figure 2.

CI(calculatedfromheadwidthandlength)(%)=headwidth/headlength×100.

CVAI was calculated from the dual cranial diagonal diameters^11^ as follows (Figure 2):

CVAI (the difference between the longer and shorter diagonals at the level of the measurement plane at an angle of 30° to the Y-axis with respect to the length of the longer 30-degree diagonal) (%) = (A-B) × 100 / A or B - whichever is greater.

Where CVAI: (%, prominent right forehead: +, prominent left forehead: -)

**Figure 3:**
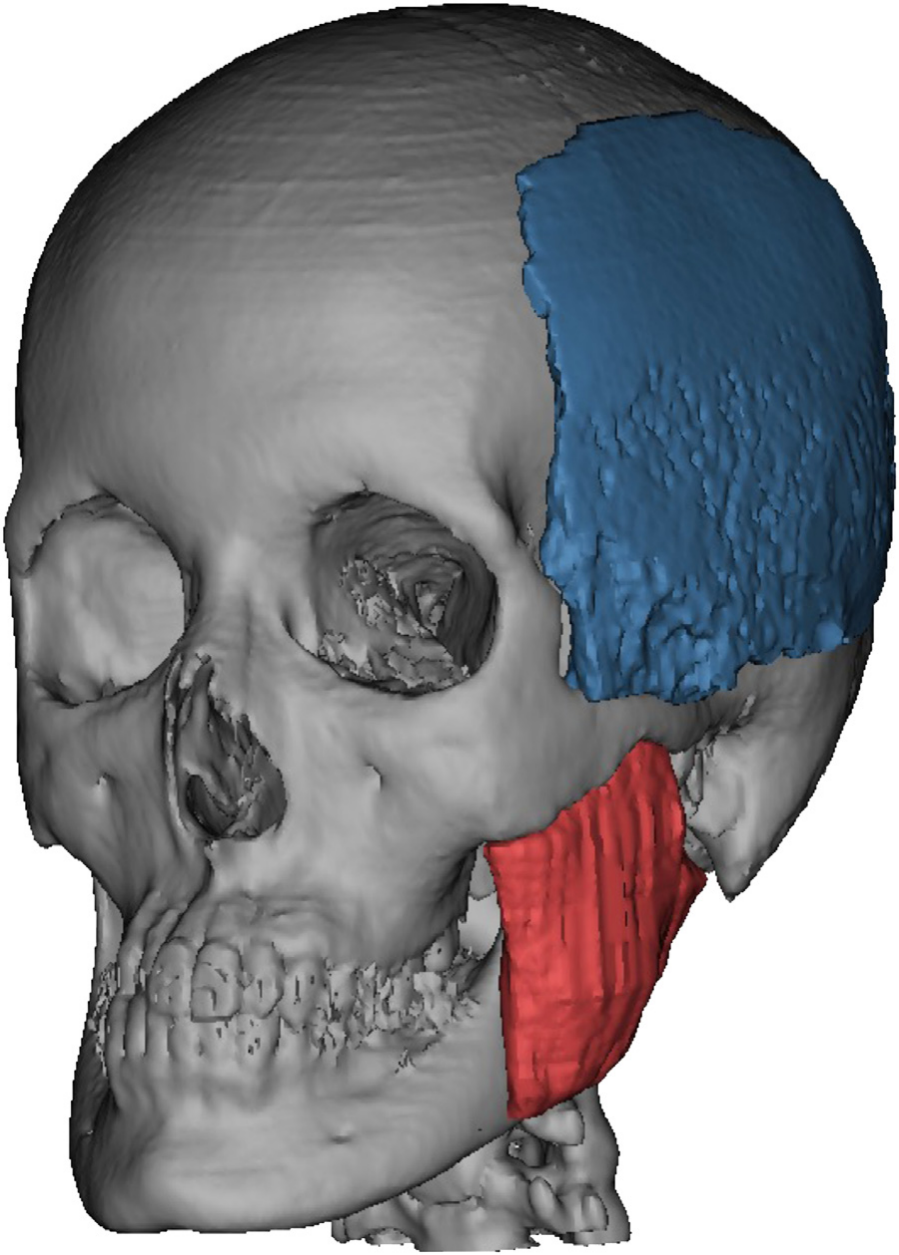
Measurement of temporal and masseter muscle volumes. The 3D temporal and masseter muscles were constructed from the CT data, and the volume was measured. Statistical analyses were performed to evaluate differences in the temporal muscle volume in the left and right muscles (variable temporal muscle volume = right temporal muscle volume - left temporal muscle volume).

The measurements of the temporalis and masseter muscles were extracted from the soft tissue CT data and their volumes were obtained using 3D construction according to our previous article^6^ (Figure 3). The volume difference between the temporalis and masseter muscles in the left and right directions was calculated (variable muscle volume = right muscle volume - left muscle volume).

### Statistical analyses

Correlations between postoperative changes in VRL-Me and factors such as age, sex, CI, CVAI, amount of surgical movement in VRL-Me, variable temporal muscle volume, and variable masseter muscle volume were analysed by univariate analyses using Pearson's correlation coefficient. The Shapiro-Wilk test was performed to validate the use of Pearson's correlation coefficient and confirmed that all parameters were normally distributed. Multivariate analysis was performed using CVAI, the amount of surgical movement in VRL-Me, and variable temporal muscle volume as predictors associated with postoperative change in VRL-Me. Multiple regression models were used to evaluate the prediction of postoperative change in VRL-Me. Differences were considered significant at *p*<0.05. Data were statistically analysed using JMP software (version 16; SAS Institute, Cary, North Carolina, USA).

## Results

**Figure 4:**
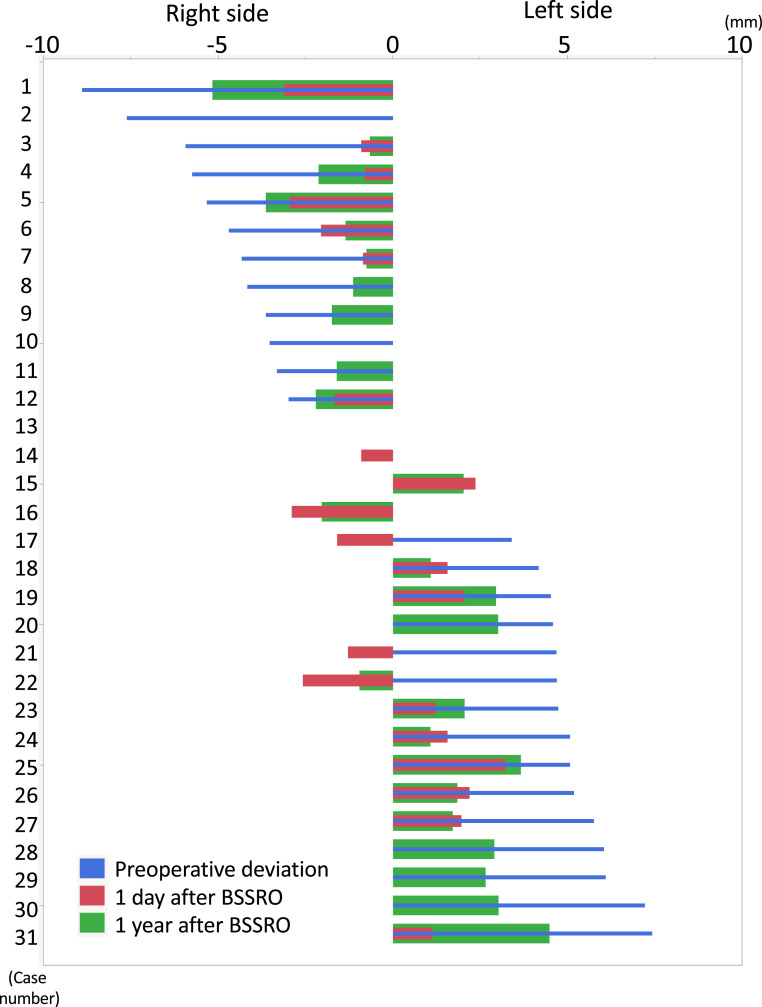
Deviation of VRL-Me (mm, right side: −, left side: +). For each of the 31 patients operated on the preoperative VRL-Me (blue bars), lateral surgical change on the day after surgery (red bars), and postoperative change during the 1-year follow-up period (green bars) are arranged according to the magnitude of the preoperative VRL-Me. Negative values indicate the right side, and positive values indicate the left side. VRL-Me, distance of vertical reference line to the menton; BSSRO, bilateral sagittal split ramus osteotomy.

In PA cephalograms, the mean preoperative deviation in VRL-Me was 0.6 ± 5.0 mm, and the amount of surgical movement in VRL-Me was 4.0 ± 1.9 mm. The mean postoperative change in VRL-Me from immediately to 1 year after surgery was 1.1 ± 1.0 mm. Seven cases (Cases No. 3, 6, 7, 18, 24, 26, and 27) of further movement towards the facial midline/opposite side (Figure 4). 3D-CT showed that the mean CVAI and CI were −0.1%±4.1% and 87.4%±5.0%, respectively. The mean temporal muscle and masseter volumes were −3746.8 ± 13,968.5 mm^3^ and 805.1 ± 3640.2 mm^3^, respectively (Table 2).

**Table 2:** Participant characteristics and posteroanterior cephalograms and three-dimensional computed tomography results.

Parameters	*n* (%)/mean±SD
Operation method: BSSRO	31
Age (y)	27.7 ± 10.5
Sex: Male	11 (35.5)
Female	20 (64.5)
CI (%)	87.4 ± 5.0
CVAI (%)	−0.1 ± 4.1
Preoperative deviation in VRL-Me (mm)	0.6 ± 5.0
Postoperative change in VRL-Me (mm)	1.1 ± 1.0
Amount of surgical movement in VRL-Me (mm)	4.0 ± 1.9
Variable temporal muscle volume (mm^3^)	−3746.8 ± 13,968.5
Variable masseter muscle volume (mm^3^)	805.1 ± 3640.2

BSSRO, bilateral sagittal split ramus osteotomy; CVAI, cranial vault asymmetry index; CI, cephalic index; VRL-Me, distance of vertical reference line to the menton.

Univariate analysis revealed a significant correlation between postoperative changes in VRL-Me and CVAI (*R* = 0.56, *p*<0.001), amount of surgical movement in VRL-Me (*R* = 0.41, *p* = 0.023), and variable temporal muscle volume (*R* = 0.37, *p* = 0.04). There was no significant correlation between the postoperative changes in VRL-Me and age, sex, CI, and the variable masseter muscle volume (Table 3).

**Table 3:** Correlation between postoperative change in VRL-Me and each independent variable by simple regression analysis.

	R	*p-*value
Age	0.029	0.92
Sex	0.31	0.094
CI	0.093	0.62
CVAI	0.56	0.001*
Amount of surgical movement in VRL-Me	0.41	0.023*
Variable temporal muscle volume	0.37	0.04*
Variable masseter muscle volume	0.16	0.38

Correlations among the postoperative change in VRL-Me, age, sex, the CI, the CVAI, the variable temporal muscle volume, and the variable masseter muscle volume were analysed using Pearson's correlation coefficient.

⁎ Statistically significant difference. CVAI, cranial vault asymmetry index; CI, cephalic index; VRL-Me, distance of vertical reference line to the menton.

Multiple regression analysis was performed using postoperative change in VRL-Me as the objective variable and CVAI, amount of surgical movement in VRL-Me, and variable temporal muscle volume as explanatory variables. All of these showed significant differences in the univariate analyses as explanatory variables. In the multivariate analysis, significant differences were observed in the CVAI (*p* = 0.003) and the amount of surgical movement in VRL-Me (*p* = 0.014; Table 4). The CVAI and the amount of surgical movement in VRL-Me were predictors of the postoperative change in VRL-Me, as determined by the multiple linear regression analyses, and thus predicted the amount of lateral recurrence after surgery. The formula used was postoperative change in VRL-Me = −0.716–0.184 *(CVAI)+0.286*(amount of surgical movement in VRL-Me) (*R* = 0.67, *p* = 0.0002).

**Table 4:** Correlation between postoperative change in VRL-Me and each independent variable by multiple linear regression analysis.

	*p-*value
CVAI	0.003*
Amount of surgical movement in VRL-Me	0.014*
Variable temporal muscle volume	0.22

Prediction of the amount of lateral deviation following BSSRO (postoperative change in VRL-Me) = −0.716–0.184*(CVAI)+0.286*(amount of surgical movement in VRL-Me).

*R* = 0.67, p-value=0.0002.

BSSRO, bilateral sagittal split ramus osteotomy; CVAI, cranial vault asymmetry index; CI, cephalic index; VRL-Me, distance of vertical reference line to the menton.

### Representative case presentation (Figure 5)

Case No. 31 is a case of both mandibular asymmetry (deviation of VRL-Me=+7.42, left-side shift) and positional plagiocephaly (CVAI=+0.7, prominent right frontal forehead). Preoperatively, orthodontic treatment was performed, followed by BSSRO (amount of surgical movement in VRL-Me=6.29 mm). At 1 day after surgery, the deviation in VRL-Me was +1.13 mm and at 1 year after surgery, the deviation was +4.48 mm, resulting in a postoperative change in VRL-Me of 3.35 mm.

**Figure 5:**
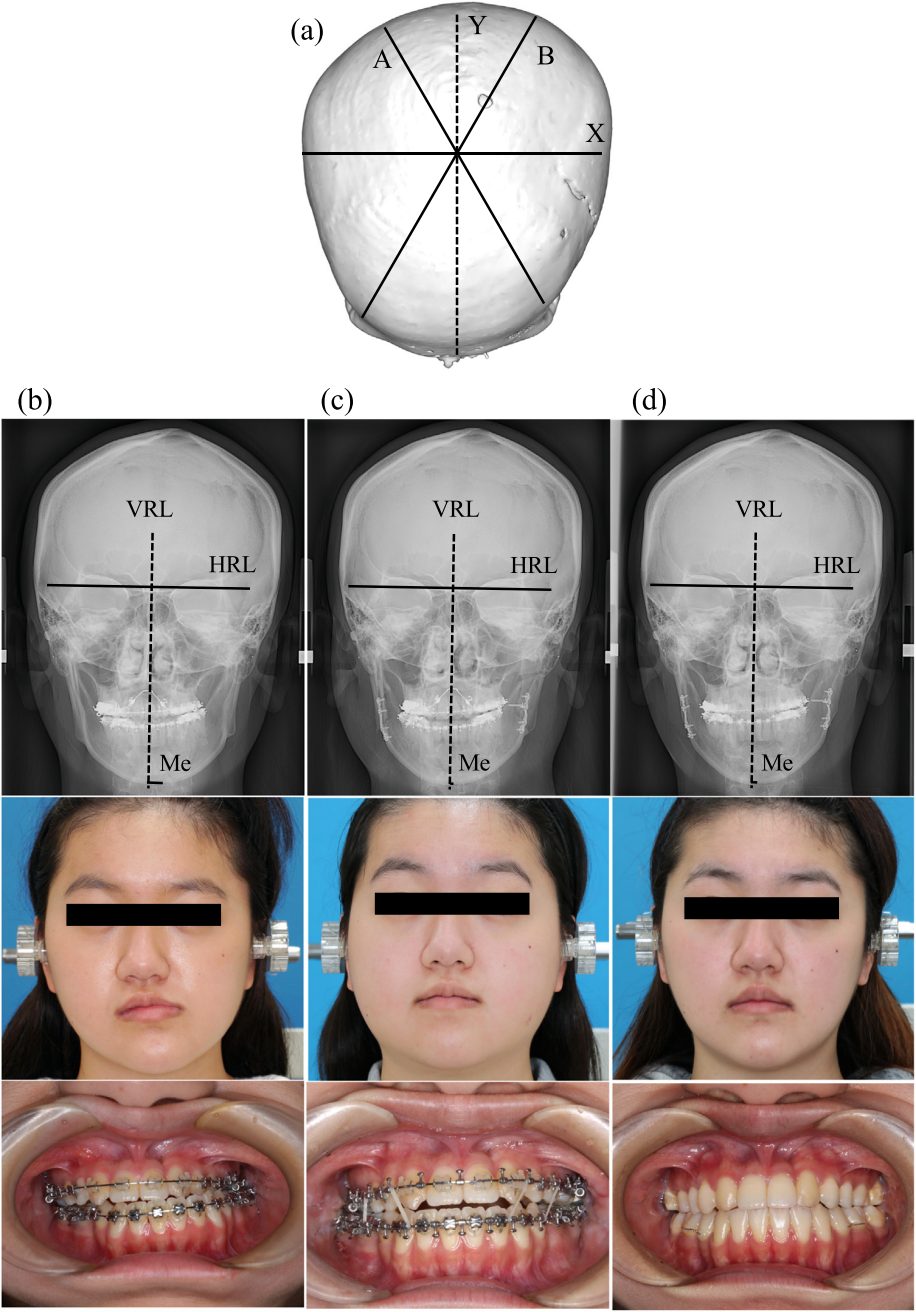
Case presentation (Case No. 31). Positional plagiocephaly based on 3D-construction image (a). Preoperative PA cephalogram, photograph of face and occlusion (b). PA cephalogram, photograph of face and occlusion at 1 day after surgery (c). PA cephalogram, photograph of face and occlusion at 1 year after surgery (d). PA, posteroanterior. VRL, vertical reference line; HRL, horizontal reference line; Me, menton; PA, posteroanterior.

## Discussion

Several studies have assessed the postoperative stability of facial asymmetry,^12^^,^^13^ all focusing on surgical techniques and none mentioning either plagiocephaly or the masticatory muscle. We previously reported that the CVAI and temporal muscle are related to the development of facial asymmetry,^6^ and we expected that both would be strongly associated with the postoperative stability of mandibular asymmetry in this study. Our results show that the relapse of mandibular asymmetry following BSSRO was clarified to be associated with CVAI and the amount of surgical movement in VRL-Me in a multiple linear regression analysis, successfully predicting the amount of lateral relapse following BSSRO.

Cranial deviation and its direction are important factors for stability following BSSRO for mandibular asymmetry with positional plagiocephaly. In other words, patients with right anterior and left protruding positional plagiocephaly posteriorly with leftward deviation and large surgical movement of the Me were prone to rightward retroversion after surgery. These results suggest that, to improve the postoperative stability following BSSRO for mandibular asymmetry, it is necessary to calculate the CVAI and amount of lateral surgical movement, to simulate the amount of postoperative relapse based on the previous predicting the amount of lateral deviation following BSSRO deviation following BSSRO analysis formula, and to consider overcorrection. Further, cranioplasty and cranial traction osteogenesis^14^^,^^15^ in childhood are expected to improve the CVAI scores and to prevent the onset of facial asymmetry and relapse after mandibular asymmetry surgery; however, more research is needed to clarify these relationships.

The temporalis muscle was not detached from the coronal process in this study. Nevertheless, temporal muscle volume—which was correlated with facial asymmetry in our previous study—correlated with relapse of asymmetry in the univariate analysis in this study but was rejected in the multiple regression analysis and thus not a useful predictor. The temporal muscle is attached to the coronoid process and has been suggested to cause lateral imbalance of the mandible in the postoperative period.^16^ Therefore, detachment of the temporal muscle from the mandible during orthognathic surgery is reportedly useful for postoperative stability.^2^ Temporal muscle did not contribute to postoperative stability in the multiple regression analysis; however, when performing BSSRO for mandibular asymmetry, intraoperative detachment of the temporal muscle from the mandibular bone is recommended if possible. Additionally, botulinum toxin administration,^17^ which is widely used for cosmetic and therapeutic purposes in the perioral and temporomandibular regions, may be useful in maintaining postoperative stability.

Conversely, the masseter muscle was not involved in lateral relapse. Stretching of the masseter and submandibular muscles due to mandibular displacement has been reported as a possible risk factor for relapse. However, the results regarding the lateral relapse were contradictory.

Several reports have indicated that the significant factor related to relapse after mandibular setback by BSSRO is the amount of mandibular setback.^18^ However, to our knowledge, previous studies have not mentioned postoperative lateral stability after BSSRO for mandibular asymmetry. In this study, we found that the amount of mandibular lateral surgical movement predicted the relapse after BSSRO for mandibular asymmetry and affected postoperative stability.

There is controversy regarding the use of resorbable and titanium plates for orthognathic surgery when assessing the stability of orthognathic surgery. To date, no difference has been reported between resorbable and titanium plates.^19^^,^^20^ In all cases of BSSRO, titanium miniplates were used. The type of plate used does not seem to affect the postoperative stability of BSSRO for mandibular asymmetry.

The limitation of this study is that it is a retrospective study with only 31 patients. However, it is remarkable that even with such a small number of patients and a small amount of lateral relapse, we were able to clarify that positional plagiocephaly affects postoperative stability following BSSRO.

On the other hand, seven cases (21.2%) showed further movement towards the facial midline/opposite side, regardless of CVAI or the amount of surgical movement. The mechanism of this direction of movement is currently unknown; in a similar study of BSSRO for asymmetric mandibular prognathism, 14 of 38 patients (36.8%) reported relapse on the ipsilateral side.^21^ Furthermore, it has been suggested that progressive condylar resorption is the most common cause of deviation after BSSRO.^22^ Therefore, we evaluated the development of progressive condylar resorption in the 31 patients in this study using cephalometric tomography. However, we found no findings suggesting progressive condylar resorption in any of the cases. In addition, the bilateral condyles were in the same position in the mandibular fossa before and after BSSRO, and no TMJ joint disorder symptoms were observed.

Positional plagiocephaly is often associated with skull base scoliosis and mandibular fossa malposition. However, in 31 cases, the CVAI was mild (−0.1 ± 4.1) and there were no ipsilateral ear malposition's on 3D-CT or bilateral condylar position discrepancies on the preoperative lateral cephalogram. Therefore, seven patients with further movement towards the facial midline/opposite side and 24 patients as control were included in a subgroup analysis. The correlation between postoperative change in VRL-Me and age, sex, CI, CVAI, surgical VRL-Me movement, temporalis muscle mass, and masseter muscle mass was analysed by univariate analysis using Pearson's correlation coefficient analysis. However, the results of the analysis indicated that there were no significant differences between the examined parameters (data not shown). Although occlusal status and adequate temporal muscle detachment may help explain the direction of deviation, in the future, we would like to accumulate a variety of cases and investigate the prevention mechanism of recurrence and mandibular asymmetry following BSSRO.

Nevertheless, to the best of our knowledge, this is the first study to show a relationship between postoperative stability of mandibular asymmetry and CVAI and the amount of lateral surgical movement. However, the underlying reasons for these correlations are not certain and require further investigation.

## Conclusions

Stability after orthognathic surgery is a significant issue. While several factors are involved in postoperative instability, this study suggests that positional plagiocephaly and the amount of surgical movement affect the lateral skeletal stability following BSSRO for facial asymmetry.

## Declaration of Competing Interest

None.
